# The effect of linagliptin on microalbuminuria in patients with diabetic nephropathy: a randomized, double blinded clinical trial

**DOI:** 10.1038/s41598-023-30643-7

**Published:** 2023-03-01

**Authors:** Mozhgan Karimifar, Jamileh Afsar, Massoud Amini, Firouzeh Moeinzadeh, Awat Feizi, Ashraf Aminorroaya

**Affiliations:** 1 Isfahan Endocrine and Metabolism Research Center, Isfahan University of Medical Sciences, Isfahan, Iran; 2 Isfahan Kidney Diseases Research Center, Isfahan University of Medical Sciences, Isfahan, Iran; 3 Department of Biostatistics and Epidemiology, School of Health, Isfahan University of Medical Sciences, Isfahan, Iran; 4 Isfahan Endocrine and Metabolism Research Center, Isfahan University of Medical Sciences, Isfahan, Iran

**Keywords:** Endocrine system and metabolic diseases, Endocrinology, Medical research

## Abstract

The aim of the present study was to investigate the effect of linagliptin on microalbuminuria in patients with diabetic nephropathy (DN). The present double-blind randomized placebo-controlled clinical trial was performed on 92 patients with DN who were divided into two groups. The intervention and control groups received linagliptin 5 mg and placebo for 24 weeks, respectively. Blood pressure, lipid profile, liver enzymes, fasting plasma glucose (FPG), and urine albumin-creatinine ratio (*UACR)* were assessed and recorded before, 12 weeks, and 24 weeks after the beginning of the intervention. The mean value of UACR decrease was significant over time in both groups, with higher decrease in linagliptin group, however, the differences between two groups were not, statistically significant (P > 0.05). However, the percentage of improvement in microalbuminuria (UACR < 30 mg/g) in the linagliptin group was significantly higher than that of the control group during 24 weeks of intervention (68.3% vs. 25%; P-value < 0.001). There was no statistically significant difference in the mean value of the UACR and other parameters between linagliptin treated and placebo treated patients with diabetic nephropathy. Further studies, with longer periods of follow-up are suggested to examine these patients’ renal outcomes.

## Introduction

Diabetes is one of the most common metabolic diseases, and its prevalence is increasing in adults, especially in developing countries such as Iran^1^. Type 2 diabetes (T2D) has been recognized as one of the most significant risk factors for microvascular and macrovascular diseases^2,3^. Diabetic nephropathy can be regarded as one of the main chronic microvascular complications in patients with T2D, occurs in about 35% of patients with diabetes, and is the most common cause of end-stage renal disease (ESRD) and death from cardiovascular diseases^4^. Diabetic nephropathy can eventually lead to chronic kidney disease (CKD) and ESRD; we also know that diabetic patients undergoing hemodialysis have more complications than non-diabetic patients undergoing hemodialysis do.The risk of cardiovascular complications is higher in diabetic patients with albuminuria. Hyperglycemia is the main pathogenesis of diabetic nephropathy. The exact mechanism of diabetic nephropathy is unknown, however, factors such as, angiotensin II, growth factors, endothelin, advanced glycation end products [AGEs]), glomerular hyper filtration or hyper perfusion lead to an increased glomerular capillary pressure and structural changes in the glomerulus^5^. Early diagnosis of microalbuminuria and its control by lowering plasma glucose and blood pressure and administering angiotensin*-*converting enzyme inhibitors or angiotensin II receptor blockers can prevent the progression of diabetic nephropathy^6^. Dipeptidyl peptidase-4 (DPP-4) inhibitors, also known as gliptins, are a novel class of oral hypoglycemic agents used to treat T2D^7,8^. These medications work by increasing the active levels of incretin peptides such as glucagon-like peptide-1 (GLP-1) and glucose-dependent insulinotropic polypeptide^9^. GLP-1 and other incretins increase the secretion of glucose-dependent insulin^10^. DPP-4, however, rapidly inactivates these peptides and reduces their effect on glucose balance. DPP-4 inhibitors increase the function of incretins by delaying their breakdown. Linagliptin is a new DPP-4 inhibitor that has been approved in 2011 as a hypoglycemic drug in the United States, Europe, and Japan^11^. Linagliptin is the only DPP-4 inhibitor that do not need dose adjustment in patient with reduced renal function. The molecular structure of this medication is based on xanthine, which is different from other DPP-4 inhibitors^10^*.* Because linagliptin has a long half-life *(more than 184 h)* and a stable inhibitory effect on DPP4, it can be administered once daily^12,13^*.* The results of previous studies indicated that linagliptin, as a monotherapy or in combination with other hypoglycemic drugs, had good safety and tolerability and improved glycemic index^14–16^. Some studies have reported that, it can improve the renal function, reduce oxidative stress, reduce glomerular sclerosis, and reduce albuminuria^17–20^. However, some other studies have not confirmed the effect of this drug on the reduction of albuminuria^21^.

Therefore, considering the limited and contradictory clinical evidence reporting the therapeutic effects of DPP-4 inhibitors, especially linagliptin, on diabetic nephropathy in patients with T2D, this study aimed to investigate the effect of linagliptin on microalbuminuria as a key step in preventing the progression of diabetic nephropathy in patients with T2D.

## Methods

### Design of the study and participants

This double-blind, randomized, placebo-controlled clinical trial was performed on 92 patients with T2D and nephropathy that referred to the Isfahan Endocrine and Metabolism Research Center from November 2019 to April 2021.

Inclusion criteria consisted of T2D patients age ≥ 18 years, and microalbuminuria (urine albumin*-*creatinine ratio (UACR) of 30–300 mg/g (in three urine samples collected consecutively over two weeks before the beginning of the study) with or without GFR reduction (less than 60)), glycated hemoglobin (HbA1c) level of 6.5–10% (48–86 mmol/mol), body mass index (BMI) of less than 40 kg/m^2^.

Furthermore, patients who were taking short-acting insulins, rosiglitazone, pioglitazone, GLP-1 receptor analogues, sodium glucose co-transporter 2 inhibitors or anti-obesity drugs within three months before the beginning of the study, those with a history of myocardial infarction, stroke, or transient ischemic attack within 6 months of the beginning of the study, patients who had non-diabetic renal failure or urinary tract infection, or those who had received a kidney transplant were not included in the study. They were excluded the study, in the case of not cooperating, not attending in the follow-up sessions, or showing drug-induced complications.

### The process of implementing interventions and measuring research variables

The study was approved by the Isfahan University of Medical Sciences ethics committee (Approval no. IR.MUI.MED.REC.1397.230), and was conducted in accordance with the Declaration of Helsinki. Written informed consent was obtained from all patients. The study protocol was registered at irct.ir as IRCT20171030037093N11 (https://irct.ir/trial/39062).

Ninety two eligible patients were selected using convenience sampling method. Then, these patients were divided into two groups using random allocation software. At the beginning of the study, patients’ demographic and clinical information including sex, age, BMI, waist circumference (WC), systolic blood pressure (SBP), diastolic blood pressure (DBP), comorbidities, duration of T2D, history of drug use (lipid-lowering drugs, hypoglycemic drugs, and antihypertensive drugs), biochemical parameters including urine creatinine (Urine Cr), urine albumin (Urine Alb), UACR, Hemoglobin A1c (HbA1c), fasting plasma glucose (FPG), serum creatinine and glomerular filtration rate (GFR) by MDRD formula, lipid profile including triglycerides (TG), high-density lipoprotein (HDL), low-density lipoprotein (LDL), and total cholesterol, and liver enzymes including alanine aminotransferase (ALT), aspartate aminotransferase (AST), and alkaline phosphatase (ALP) were recorded.

For all patients, a routine diabetes treatment was prescribed according to the standard protocol of American Diabetes association (ADA) statement. In addition, 5 mg of linagliptin was administered to patients in the intervention group for 24 weeks while patients in the control group received placebo for 24 weeks.

It should be mentioned that in order to comply with the double-blind condition, linagliptin and placebo which had been already prepared by Alhavi Pharmaceutical Company, located in Tehran, Iran, in the same shape, size, and color. Starch had been used to make placebo tablets. The prepared drugs had been coded, and provided to the researcher. Therefore, the researcher, patients, the information evaluator, and the statistical analyst had no knowledge of the type of the intervention performed in the two groups.

Furthermore, all patients were requested to follow the healthy dietary patterns and proper physical activity in the treatment process to control these factors as much as possible and prevent the disruptive effect of patients’ eating habits and physical activity on the results of the study.

Patients were evaluated in terms of the complications of the medication two weeks after the beginning of the intervention and then every 4 weeks. In addition, patients’ anthropometric, blood pressure, and biochemical factors were assessed 12 weeks and 24 weeks after the intervention. To perform accurate measurements and evaluations, data collection was performed by a single specialist technician, and all biochemical tests were performed only in the Laboratory of Isfahan Endocrine and Metabolism Research Center.

### Statistical analysis

The collected data were analyzed by SPSS software (ver.26) (IBM SPSS Statistics for Windows, Armonk, NY: IBM Corp.). Quantitative and qualitative variables were reported as means ± standard deviation (SD) and number (percentage), respectively. Kolmogorov–Smirnov test and Q-Q diagram were used to check the normality of data distribution. Basic quantitative and qualitative variables of study participants were compared two groups using independent samples *t*-test and Chi-squared test, respectively. Moreover, one-way repeated measures analysis of variance (ANOVA) was used for intra-group and inter-group comparisons to evaluate the mean changes of quantitative variables over 24 weeks from the beginning of the intervention. Mauchly’s test was used to evaluate the sphericity hypothesis, and if it was not established, the multivariate analysis was used. We used Bonferroni post hoc test for doing pairwise comparisons between time points as well as for comparing two groups at each time point. The significance level of less than 0.05 was considered in all analyses.

### Ethical approval

All procedures performed in studies involving human participants were in accordance with the ethical standards of the institutional and/or national research committee and with the 1964 Helsinki declaration and its later amendments or comparable ethical standards. The study was approved by the ethics Committee of the Isfahan University of Medical Sciences (Approved code: IR.MUI.MED.REC.1397.230) and its clinical trial code is recorded (IRCT20171030037093N11).

### Informed consent

Written informed consent was obtained from all patients for precipitation and registration.

## Results

**Figure 1:**
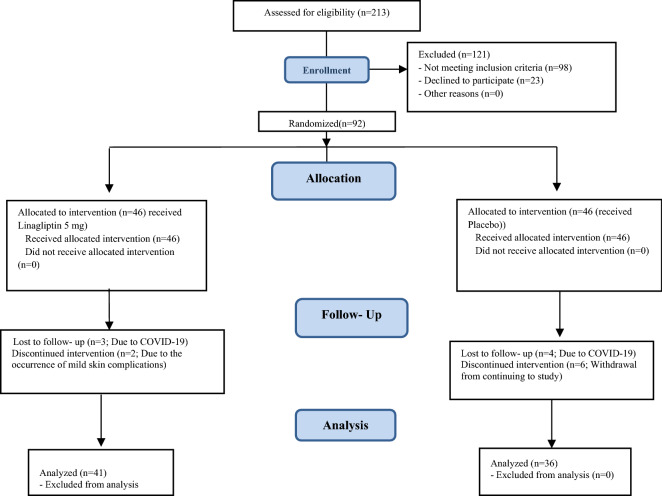
Consort flow diagram for recruitment of patients.

Among 213 eligible patients, 92 patients were finally enrolled (46 patients in linagliptin group and 46 patients in control group). Five patients from the linagliptin group (3 patients due to their confirmed COVID-19 infection and 2 patients due to their mild skin complications had withdrawal from the study) and 10 patients from the control group (4 patients due to their confirmed COVID-19 infection and 6 patients due to their non-attendance in subsequent follow-ups) were excluded from the study (Fig. 1). Finally, 41 patients in the linagliptin group and 36 patients in the control group continued the study until the end of the trial. Basal characteristics of both groups are mentioned in Table 1. There was no significant difference between the two groups in terms of age, sex, BMI, comorbidities, and used medications (Table 1).

**Table 1:** Patients’ basic characteristics in the two groups.

Characteristics	Linagliptin group (n = 41)	Control group (n = 36)	P-value**
Age; year	57.56 ± 6.55	57.53 ± 5.77	0.981
Sex			
Male	11 (26.8%)	11 (30.6%)	0.718
Female	30 (73.2%)	25 (69.4%)	
BMI; kg/m^2^	30.39 ± 17.55	29.76 ± 4.75	0.865
Comorbidity			
None	19 (46.3%)	12 (33.4%)	0.281
HTN	21 (51.2%)	21 (58.3%)	
Hypothyroidism	1 (2.5%)	3 (8.3%)	
Medication*			
Anti-diabetic	26 (63.4%)	23 (63.9%)	
Basal insulin	15 (36.6%)	13 (36.1%)	0.966
ACEI	3 (7.3%)	5 (13.9%)	
ARB	28 (68.3%)	23 (63.9%)	
B-blocker	5 (12.2%)	3 (8.3%)	
CCB	2 (4.9%)	5 (13.9%)	
Diuretic	3 (7.3%)	3 (8.3%)	
Statin	35 (85.4%)	30 (83.3%)	
Aspirin	25 (64.1%)	19 (52.8%)	

Data are shown as n (%) or mean ± SD for categorical and continuous variables, respectively.

*ACEI* angiotensin-converting enzyme inhibitors, *ARB* angiotensin receptor blockers, *CCB* calcium channel blockers.

*Each patient might take more than one drug.

**Resulted from independent samples t-test for continuous and chi-squared test for categorical variables.

The patients’ mean weight, WC, and BMI did not differ significantly between the two groups before, 12 weeks, and 24 weeks after the intervention (Table 2). The changes in anthropometric variables were not significant over time in any of the groups (P_Time_ > 0.05) and between the two groups. Furthermore, mean values of anthropometric measures were not significantly different between the two groups in any follow up time points (P_group_ > 0.05). Moreover, the interactive effect of time and intervention was not significant (P_time*group_ > 0.05) (Table 2).

**Table 2:** Patients’ anthropometric parameters in two groups.

Variables	Baseline	12 weeks	24 weeks	Repeated measures ANOVA		
				P_time_	P_group_	P_time*group_
Weight; kg						
Linagliptin group (n = 41)	74.66 ± 13.64	74.76 ± 13.97	74.19 ± 13.44	0.290	0.667	0.302
Control group (n = 36)	75.24 ± 11.32	75.67 ± 10.93	76.44 ± 10.78	0.447		
P	0.751	0.759	0.437			
WC; cm						
Linagliptin group (n = 41)	98.80 ± 18.74	98.86 ± 18.52	98.16 ± 18.20	0.501	0.932	0.528
Control group (n = 36)	98.80 ± 14.14	99.50 ± 14.51	96.23 ± 20.19	0.291		
P	0.885	0.872	0.719			
BMI; kg/m^2^						
Linagliptin group (n = 41)	30.39 ± 17.55	27.95 ± 5.12	30.72 ± 16.72	0.533	0.917	0.613
Control group (n = 36)	29.76 ± 4.75	30.03 ± 4.47	29.90 ± 4.26	0.305		
P	0.865	0.071	0.780			

Data are shown as mean ± SD.

*WC* waist circumference, *BMI* body mass index.

P is obtained from an independent samples *t*-test conducted for comparing the mean of the variables between the two groups at each follow-up time point. P_group_ shows the overall difference between the two groups over the follow-up period. P_time_ indicates the changes in the mean of the variables in the intervention and control groups over the follow-up period. P_time*group_ presents the interaction between the time and intervention. P_group_, P_time_, and P_time*group_ are obtained from repeated measures ANOVA.

The results of the repeated measures ANOVA revealed that although SBP (P_Time_ = 0.021) and DBP (P_Time_ = 0.010) decreased significantly over 24 weeks after the beginning of the intervention in the linagliptin group, however its changes were not significantly different between the two groups over time (P_group_ > 0.05). We did not observe significantly interactive effect of time and intervention Ptime_*group_ = 0.324) (Table 3).

**Table 3:** Parameters of diabetic patient's blood pressure, plasma glucose, lipid profile, and liver enzymes in linagliptin and placebo treated groups.

Variables	Baseline	12 weeks	24 weeks	Repeated measures ANOVA		
				P_time_	P_group_	P_time*group_
SBP; mmHg						
Linagliptin group (n = 41)	125.67 ± 8.51	122.84 ± 7.41	121.35 ± 6.84	**0.021**^**#**^	0.122*	0. 324*
Control group (n = 36)	117.48 ± 21.59	121.28 ± 6.10	122.71 ± 7.98	0.195		
P	**0.021**^**#**^	0.337	0.438			
DBP; mmHg						
Linagliptin group (n = 41)	77.32 ± 7.91	75.27 ± 6.45	72.16 ± 7.50	**0.010**^**#**^	0.826*	0.273*
Control group (n = 36)	72.36 ± 10.31	73.14 ± 6.31	72.71 ± 6.68	0.962		
P	**0.020**^**#**^	0.162	0.743			
FPG; mg/dL						
Linagliptin group (n = 41)	156.63 ± 39.46	145.97 ± 33.97	136.89 ± 31.48	** < 0.001**^**#**^	0.839	0.670
Control group (n = 36)	153.94 ± 34.47	144.03 ± 34.79	138.83 ± 34.01	**0.003**^**#**^		
P	0.753	0.811	0.803			
HbA1c; %						
Linagliptin group (n = 41)	8.17 ± 1.02	7.88 ± 1.10	7.54 ± 1.03	** < 0.001**^**#**^	0.893	0.921
Control group (n = 36)	8.22 ± 1.10	7.81 ± 1.21	7.54 ± 1.30	** < 0.001**^**#**^		
P	0.806	0.799	0.999			
TG; mg/dL						
Linagliptin group (n = 41)	195.05 ± 67.05	180.24 ± 50.61	162.97 ± 46.29	**0.01**^**#**^	0.141	0.346
Control group (n = 36)	213.47 ± 96.23	196.37 ± 80.93	197.74 ± 85.01	0.309		
P	0.328	0.311	**0.033**^**#**^			
Cholesterol; mg/dL						
Linagliptin group (n = 41)	175.75 ± 31.76	168.75 ± 33.18	166.97 ± 35.33	0.082	0.536	0.519
Control group (n = 36)	178.41 ± 39.97	173.51 ± 34.19	175.54 ± 36.68	0.665		
P	0.746	0.551	0.316			
LDL; mg/dL						
Linagliptin group (n = 41)	87.66 ± 24.02	80.05 ± 26.12	86.98 ± 24.02	0.080	0.632	0.548
Control group (n = 36)	87.57 ± 26.52	84.54 ± 19.46	90.48 ± 22.81	0.285		
P	0.988	0.413	0.528			
HDL; mg/dL						
Linagliptin group (n = 41)	45.22 ± 10.15	44.81 ± 9.45	43.73 ± 10.47	0.690	0.750	0.083
Control group (n = 36)	41.64 ± 8.45	45.57 ± 10.11	44.51 ± 9.59	**0.013**^**#**^		
P	0.100	0.742	0.743			
ALT; U/L						
Linagliptin group (n = 41)	18.73 ± 9.31	22.02 ± 10.91	21.13 ± 11.03	0.08	0.553	0.906
Control group (n = 36)	20.64 ± 9.62	23.40 ± 10.22	21.97 ± 10.60	0.169		
P	0.380	0.584	0.744			
AST; U/L						
Linagliptin group (n = 41)	21.48 ± 6.86	21.19 ± 7.60	21.78 ± 9.84	0.841	0.603	0.941
Control group (n = 36)	22.66 ± 7.31	22.34 ± 8.68	22.63 ± 9.72	0.941		
P	0.468	0.550	0.715			
ALP; U/L						
Linagliptin group (n = 41)	226.44 ± 57.10	235.38 ± 50.68	230.243 ± 65.30	0.751	**0.032**^**#**^	0.992
Control group (n = 36)	205.97 ± 46.16	211.23 ± 42.95	205.653 ± 46.46	0.473		
P	0.091	**0.033**^**#**^	0.071			

*SBP* systolic blood pressure, *DBP* diastolic blood pressure, *FPG* fasting plasma glucose, *HbA1C* hemoglobin A1c, *TG* triglycerides, *LDL* low-density lipoprotein, *HDL* high-density lipoprotein, *ALT* alanine aminotransferase, *AST* aspartate aminotransferase, *ALP* alkaline phosphatase.

P is obtained from an independent samples *t*-test conducted for comparing the mean of the variables between the two groups at each follow-up time point. P_group_ indicates the overall difference between the two groups over the follow-up period. P_time_ shows the changes in the mean of the variables in the intervention and control groups separately over the follow-up period. P_time*group_ demonstrates the interaction between the time and intervention. P_group_, P_time_, and P_time*group_ was obtained from repeated measures ANOVA.

Significant values are given in bold.

^#^Statistically significant results.

*If the baseline values had a significant difference between the two groups, it was considered as a confounder and it was adjusted in the repeated measures ANOVA.

Although, the mean values of FPG, HbA1c, and TG (not significantly decreased in control group) in each of two groups decreased significantly over follow up time (P_time_ < 0.05), however the mean change of these variables were not significantly different between two groups (P_group_ > 0.05). The interactive effect of time and intervention was not statistically significant (P_time*group_ > 0.05). Also, the mean value of FPG and HbA1c was not significantly different between two groups in none of follow up time point (P > 0.05) and mean value of TG at 24 weeks after intervention in linagliptin group was significantly lower than control group (P < 0.05) (Table 3).

Other parameters of lipid profile and liver enzymes were not found to be significantly different in inter-group (P_time_ > 0.05) and intra-group comparisons (P > 0.05). Twelve weeks after intervention, only the liver enzyme ALP in the linagliptin group with the mean of 235.38 ± 50.68 U/L was significantly higher than the control group with the mean of 211.23 ± 42.95 U/L. Therefore, the results of the repeated measures ANOVA indicated that the effect of intervention (P_group_ = 0.032) was significant in this variable, and the interactive effect of time and intervention (P_time*group_ > 0.05) was not statistically significant (Table 3).

Finally, none of the parameters related to the renal function and microalbuminuria were significantly different between the two groups before, 12 weeks, and 24 weeks after the intervention (P > 0.05). However, GFR decrease and Cr increase were significant in the linagliptin group over time (P_time_ < 0.05). But the mean change of these variables were not significantly different between two groups (P_group_ > 0.05) and the interactive effect of time and intervention (P_time*group_ > 0.05) also was not significant (Table 4).

**Table 4:** Parameters related to renal function and microalbuminuria in two groups.

Variables	Baseline	12 weeks	24 weeks	Repeated measures ANOVA		
				P_time_	P_group_	P_time*group_
GFR						
Linagliptin group (n = 41)	68.44 ± 11.71	65.12 ± 11.15	63.55 ± 9.91	**0.034**^**#**^	0.320	0.632
Control group (n = 36)	65.22 ± 12.16	62.32 ± 12.07	62.51 ± 11.29	0.238		
P	0.241	0.309	0.679			
Cr; mg/dl						
Linagliptin group (n = 41)	0.93 ± .0.14	0.97 ± 0.12	0.99 ± 0.13	**0.035**^**#**^	0.715	0.804
Control group (n = 36)	1.00 ± 0.17	1.02 ± 0.19	1.03 ± 0.14	0.371		
P	0.058	0.178	0.196			
Urine Cr; g/dl						
Linagliptin group (n = 41)	85.44 ± 34.22	91.15 ± 30.90	94.91 ± 47.04	0.627	0.227	0.129
Control group (n = 36)	90.55 ± 37.80	78.81 ± 36.70	79.51 ± 37.81	0.101		
P	0.535	0.127	0.132			
Urine Alb; mg/dl						
Linagliptin group (n = 41)	7.47 ± 4.49	3.98 ± 2.07	2.72 ± 2.72	** < 0.001**^**#**^	0.990	**0.027**
Control group (n = 36)	6.52 ± 5.04	4.29 ± 3.16	3.45 ± 3.01	** < 0.001**^**#**^		
P	0.384	0.616	0.285			
UACR; mg/g						
Linagliptin group (n = 41)	86.59 ± 47.68	45.93 ± 26.27	31.29 ± 31.69	**< 0.001**^**#**^	0.778	**< 0.001**
Control group (n = 36)	74.02 ± 43.64	54.93 ± 33.96	45.49 ± 30.55	**< 0.001**^**#**^		
P	0.234	0.211	0.057			

*Cr* creatinine, *GFR* glomerular filtration rate test, *UrineAlb* urine albumin, *UrineCr* urine creatinine, *UACR* urine albumin-creatinine ratio.

P is obtained from an independent samples *t*-test conducted for comparing the mean of the variables between the two groups at each follow-up time point. P_group_ indicates the overall difference between the two groups over the follow-up period. P_time_ demonstrates the changes in the mean of the variables in the intervention and control groups over the follow-up period. P_time*group_ indicates the interaction between the time and intervention. P_group_, P_time_, and P_time*group_ are obtained from repeated measures ANOVA.

Significant values are given in bold.

^**#**^Statistically significant results.

In addition, although the decrease in urine albumin and UACR was significant in both groups over time (P_time_ < 0.05), however, the mean change of these variables was not significantly different between two groups (P_group_ > 0.05). The interactive effect of time and intervention was significant in these two variables (P_time*group_ < 0.05); (Table 4). Mean values of the renal function and microalbuminuria were not significantly different in each follow up time points (P > 0.05).

**Figure 2:**
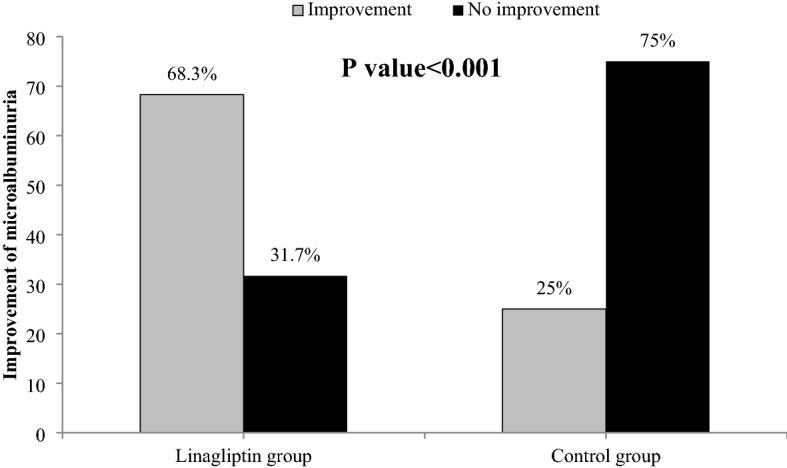
Percentage of improvement of microalbuminuria (UACR < 30 mg/dl) in the two groups.

The percentage of improvement in microalbuminuria (UACR < 30) in the linagliptin group was significantly higher than that of the control group during 24 weeks of intervention (68.3% vs. 25%; P-value < 0.001) (Fig. 2).

It should be noted that linagliptin adverse effects such as hypoglycemia or acute pancreatitis were not observed.

## Discussion

Since there were several studies on the effectiveness or ineffectiveness of linagliptin on albumin excretion in urine^21,24^, we decided to investigate this issue in a controlled study. In our study, linagliptin did not cause a significant decrease in urinary albumin excretion in diabetic patients compared to the control group. The results of a recent human study also indicated that linagliptin had no significant effect on reducing albuminuria. The duration of this study was 24 weeks like our study. These researchers believe that a longer-term treatment is needed to determine the renal effects of this drug^21^. In our study, according to the interactive effect of time and intervention, and the greater improvement of UACR (< 30 mg/g), in the linagliptin group (68.3%) than in the control group (25%) (Fig. 2), it seems that we need a longer intervention to investigate the effect of linagliptin on albuminuria. Interestingly, other studies have reported the non-albuminuric protective effects of linagliptin^22,23^. Therefore, more research is required to elucidate the renal biology and pathophysiology of DPP-4 in this regard.

On the other hand, FPG and HbA1C had a significant decrease in both linagliptin and control groups. A study, conducted by Ito et al. indicated that the mean level of HbA1C in the two groups with and without receiving linagliptin was not significantly different^24^. In another study, the levels of HbA1C were not significantly different between the intervention and placebo groups^25^. In contrast with the results of our study, Groop et al. research revealed that the linagliptin significantly improved the glycemic control in patients with type-2 DM^21^. However, the effect of other factors such as participants' degree of adherence to the diabetic diet and their level of physical activity cannot be ignored as they may play a role in controlling plasma glucose.

The results of the present study revealed that triglyceride were significantly reduced in each group over 24 weeks from the beginning of the intervention, however the decreases were not significantly different between two groups. Howevere, the results of Monami et al. study showed that DPP-4 inhibitors had a reducing effect on triglycerides^26^. According to a meta-analysis, a reducing effect of combination therapy of DPP-4 inhibitors and metformin on triglycerides and cholesterol was reported^27^.

In the present study, cholesterol change was not significantly different between the two groups.

In addition to our study, among liver enzymes, ALP was significantly higher in the linagliptin group as compared with the control group 12 weeks after the intervention. In this respect, the results of the repeated measures ANOVA indicated the significant effect of the intervention. It is important to note that the increase in ALP was not significant between two groups 24 weeks after the beginning of the intervention. The reason for the transient increase in alkaline phosphatase in the twelfth week of the study in the linagliptin group may be due to the effect of linagliptin on bone metabolism or indicate hepatic cholestasis. As we had not measured GGT (Gama-Glutamyl Transferase), we cannot determine the alkaline phosphataseʼ origin. However, the effects of DPP-4 inhibitors, including linagliptin, on bone metabolism are still unknown. One study by Kanda et al. showed a protective effect of linagliptin on the bones of diabetic rats^28^. However, as the increase in ALP was not significant between the two groups 24 weeks after the beginning of the intervention, it seems that the increased in ALP is not an important issue.

The results of the evaluation of renal function factors as well as microalbuminuria also showed that although there was a significant decrease in GFR and a significant increase in Cr in the linagliptin group, the effect of the intervention and the interactive effect of time and intervention were still not significant. Moreover the rate of decrease in GFR and increase in creatinine was not more than 30% and the effect of the intervention was not significant, it is necessary to examine this finding with more participants and longer follow-up period. In this regard, Nishida et al. conducted a study to investigate the effects of DPP-4 inhibition on diabetic patients and noted a negative brief effect on creatinine^23^. However, a study by Rosenstock et al. found that linagliptin was safe for the kidneys^12^.

It should be noted that this study was also associated with some limitations and strengths. The investigation area of the present study, which was the evaluation of the effect of linagliptin on microalbuminuria in patients with diabetic nephropathy, can be considered novel as few studies have been performed with this aim. The mentioned point can be regarded as one of the strong points of this study. However, this study was only been performed on patients with diabetic nephropathy (microalbuminuria with mild reduced GFR), so it is imprecise whether the findings can be generalized to patients with more advanced diabetic kidney diseases. In addition, the small number of participants and the short follow-up period can be regarded as another limitation of this study.

## Conclusion

Linagliptin did not cause a significant decrease in urinary albumin excretion in diabetic patients with nephropathy compared to the control group, however, the percentage of improvement in microalbuminuria (UACR < 30 mg/g) in the linagliptin group was significantly higher than that of the control group during 24 weeks of intervention. We recommend more studies with longer periods of follow-up to examine the renal outcome of linagliptin in patients with diabetic nephropathy.

## Data Availability

The data that support the findings of this study are available on request from the corresponding author, A. A. The data are not publicly available due to their containing information that could compromise the privacy of research participants.
